# Supplementary material for: Metabolite Predictors of Breast and Colorectal Cancer Risk in the Women’s Health Initiative
Source: Metabolites. 2024 Aug 20;14(8):463. doi: 10.3390/metabo14080463 (PMC11356420; doi:10.3390/metabo14080463)
Supplement: Supplementary file 1 [file metabolites-14-00463-s001.zip › Supplemental materials.pdf]

SUPPLEMENTAL METHODS

Imputing missing data

In this section, we describe the procedure we used to create our final datasets for each analysis considered. We used multiple imputation via chained equations (MICE, implemented in the R package mice; [1-2] to perform imputations. For each missing value of a clinical covariate or metabolite, we used all observed values of the remaining variables excluding the outcome in the imputation model. By not using the outcome variable to perform imputations, we can impute the data outside of our algorithm for estimating the performance of our procedure (see, e.g., Jaeger et al.[3]). Imputations were performed using a maximum of ten iterations and predictive mean matching [1] where possible; if the predictive mean matching algorithm failed to converge (which occurred for several platforms), we used tree-based imputation [1].

Our final analysis dataset was the result of two steps. We first created a common set of complete-data covariates for all analyses by performing a single round of MICE to impute any missing values of the demographic/clinical variables. Then, in an effort to minimize the effect of possibly correlated metabolites on our results and to study the utility of different platforms, we imputed missing metabolites for each platform separately. For each platform (besides NMR, which had no missing data), we created two datasets: one using 10 rounds of MICE based on only the base set of covariates, and the second using 10 rounds of MICE based on the base-set and complete-data clinical covariates. We used the same set of covariates for both imputation and regression in an effort to harmonize the analyses.

Variable selection procedures

Here, we provide further details on the algorithms we used for variable selection. For the lasso, we used ten-fold cross-validation to select the tuning parameter, and forced the base set of covariates into all models. Our approach to Super Learner-based variable selection involved several steps. We first computed a variable importance measure for each candidate algorithm: estimated coefficient for the elastic net and decrease in Gini impurity for both trees and forests. We then ranked the variables from most to least important by the algorithm-specific metrics and combined the ranks using the convex weights of the Super Learner. We then selected variables with weighted rank in the top 20. This ensures that algorithms with high weight in the Super Learner ensemble – implying that the algorithm has favorable cross-validated performance – have large influence in selecting variables.

Assessing prediction performance

After selecting a set of metabolites and covariates, it is of interest to assess the performance of these variables in predicting either BC or CRC. Assessing panel performance is complicated by the initial variable selection step and by the multiple imputations. As discussed in the main manuscript, prediction performance assessment following variable selection requires care [4]. To address this, we performed cross-fitting within Monte-Carlo sampling on only one imputed dataset (to limit computation time) for each combination of outcome, platform, and set of covariates (base set-only and all covariates); this provides an unbiased assessment of the entire procedure, from variable selection to prediction. We used only a single imputed dataset for this portion of the analysis due to computation time. More specifically, for each of 100 replicates, we performed the following procedure:

1. generate a random vector $B_n \in \{1, \dots, 5\}^n$ by sampling uniformly from $\{1, \dots, 5\}$ with replacement, and for each $v \in \{1, \dots, 5\}$, denote by D_v the data with index in $\{i: B_{n,i} = v\}$;
2. for $v \in \{1, \dots, 5\}$:
    a. obtain a panel of metabolites and covariates using the desired variable selection procedure on the training data $\bigcup_{j \neq v} D_j$;
    b. train a regression algorithm on the training data $\bigcup_{j \neq v} D_j$ and only the selected variables;
    c. predict on the withheld test data D_v and measure performance of the prediction algorithm using AUC;
3. compute the cross-validated AUC (CV-AUC) over the five train/test splits, along with an estimate of the variance of the estimated CV-AUC [5].

After termination, we average over the 100 Monte-Carlo replicates to obtain a point estimator of the performance of our procedure and obtain a Wald-type 95% confidence interval (CI) using the average variance.

SUPPLEMENTAL TABLES

Supplemental Table S1. Candidate learners in the Super Learner ensemble along with their R implementation, tuning parameter values, and description of the tuning parameters. All tuning parameters besides those listed here are set to their default values. In particular, the random forests are grown with 500 trees, a minimum node size of 5 for continuous outcomes and 1 for binary outcomes, and a subsampling fraction of 0.632; the boosted trees are grown with a maximum of 1000 trees, shrinkage rate of 0.1, and a minimum of 10 observations per node.

Candidate Learner	R Implementation	Tuning parameter and possible values	Tuning parameter description
Random forests	ranger (Wright and Ziegler, 2017)	$mtry = \sqrt{p}^{\dagger}$	Number of variables to possibly split at in each node
Gradient boosted trees	xgboost (Chen et al., 2020)	$max.depth \in \{1, 4\}$	Maximum tree depth
Elastic net	glmnet (Friedman et al., 2010)	mixing parameter $\alpha \in \{0, 0.25, 0.5, 0.75, 1\}$	Trade-off between l_1 and l_2 regularization‡

†: p denotes the total number of predictors.

‡: $\alpha = 0$ denotes ridge regression, while $\alpha = 1$ denotes lasso regression.

Supplemental Table S2. Cross-validated area under the receiver operating characteristic curve (CV-AUC) averaged over 100 Monte-Carlo replications of each variable selection + regression procedure for predicting breast cancer, with 95% confidence intervals (CIs). An analysis with only covariates (base set + clinical) is provided for comparison. The library of candidate learners used in the Super Learner (SL) is provided in Supplemental Table S1.

**Variables**	**Variable selection procedure**	**Prediction procedure**	**CV-AUC [95% CI]**
Base set of covariates and demographic/clinical	Lasso	GLM	0.569 [0.567, 0.571]
		SL	0.570 [0.568, 0.572]
	None	SL	0.568 [0.567, 0.57]
		SL (with screens)	0.572 [0.57, 0.573]
	SL	GLM	0.569 [0.567, 0.571]
		SL	0.569 [0.567, 0.571]
GC-MS metabolites			
+ base set of covariates	Lasso	GLM	0.470 [0.468, 0.472]
		SL	0.494 [0.491, 0.497]
	None	SL	0.494 [0.491, 0.497]
		SL (with screens)	0.486 [0.483, 0.489]
	SL	GLM	0.469 [0.467, 0.471]
		SL	0.493 [0.49, 0.496]
+ base set of and demographic/clinical covariates	Lasso	GLM	0.540 [0.538, 0.542]
		SL	0.541 [0.539, 0.543]
	None	SL	0.550 [0.548, 0.552]
		SL (with screens)	0.548 [0.546, 0.55]
	SL	GLM	0.540 [0.538, 0.542]
		SL	0.542 [0.54, 0.544]
LC-MS metabolites			

+ base set of covariates	Lasso	GLM	0.472 [0.47, 0.474]
		SL	0.494 [0.491, 0.497]
	None	SL	0.499 [0.497, 0.501]
		SL (with screens)	0.499 [0.497, 0.501]
	SL	GLM	0.477 [0.476, 0.479]
		SL	0.496 [0.493, 0.498]
+ base set and demographic/clinical covariates	Lasso	GLM	0.559 [0.557, 0.561]
		SL	0.560 [0.558, 0.562]
	None	SL	0.562 [0.56, 0.564]
		SL (with screens)	0.563 [0.561, 0.565]
	SL	GLM	0.559 [0.557, 0.561]
		SL	0.560 [0.558, 0.562]
Lipidyzer metabolites			
+ base set of covariates	Lasso	GLM	0.516 [0.514, 0.518]
		SL	0.524 [0.521, 0.526]
	None	SL	0.538 [0.536, 0.54]
		SL (with screens)	0.536 [0.534, 0.538]
	SL	GLM	0.508 [0.507, 0.51]
		SL	0.517 [0.515, 0.52]
+ base set and demographic/clinical covariates	Lasso	GLM	0.567 [0.565, 0.569]
		SL	0.569 [0.567, 0.571]
	None	SL	0.574 [0.572, 0.576]
		SL (with screens)	0.575 [0.573, 0.576]
	SL	GLM	0.557 [0.555, 0.559]
		SL	0.561 [0.559, 0.562]
NMR metabolites			
+ base set of covariates	Lasso	GLM	0.472 [0.47, 0.474]

+ base set and demographic/clinical covariates	None<br>SL<br>Lasso<br>None<br>SL	SL<br>SL<br>SL (with screens)<br>GLM<br>SL<br>GLM<br>SL<br>SL<br>SL (with screens)<br>GLM<br>SL	0.495 [0.492, 0.498]<br>0.489 [0.487, 0.491]<br>0.487 [0.485, 0.49]<br>0.478 [0.476, 0.48]<br>0.497 [0.494, 0.499]<br>0.560 [0.559, 0.562]<br>0.562 [0.561, 0.564]<br>0.559 [0.558, 0.561]<br>0.565 [0.563, 0.566]<br>0.561 [0.559, 0.563]<br>0.562 [0.56, 0.564]

Supplemental Table S3. Cross-validated area under the receiver operating characteristic curve (CV-AUC) averaged over 100 Monte-Carlo replications of each variable selection + regression procedure for predicting colorectal cancer, with 95% confidence intervals (CIs). An analysis with only covariates (base set + clinical) is provided for comparison. The library of candidate learners used in the Super Learner (SL) is provided in Supplemental Table S1.

**Variables**	**Variable selection procedure**	**Prediction procedure**	**CV-AUC [95% CI]**
Base set and demographic/clinical covariates	Lasso	GLM	0.541 [0.537, 0.544]
		SL	0.533 [0.53, 0.537]
	None	SL	0.553 [0.55, 0.557]
		SL (with screens)	0.558 [0.555, 0.561]
	SL	GLM	0.544 [0.541, 0.548]
		SL	0.537 [0.533, 0.541]
GC-MS metabolites			
+ base set of covariates	Lasso	GLM	0.514 [0.51, 0.517]
		SL	0.492 [0.488, 0.496]
	None	SL	0.490 [0.486, 0.494]
		SL (with screens)	0.492 [0.488, 0.495]
	SL	GLM	0.514 [0.511, 0.517]
		SL	0.491 [0.487, 0.496]
+ base set and demographic/clinical covariates	Lasso	GLM	0.524 [0.521, 0.528]
		SL	0.521 [0.517, 0.525]
	None	SL	0.520 [0.517, 0.524]
		SL (with screens)	0.522 [0.518, 0.525]
	SL	GLM	0.526 [0.523, 0.53]
		SL	0.524 [0.52, 0.527]
LC-MS metabolites			

+ base set of covariates	Lasso	GLM	0.584 [0.581, 0.588]
		SL	0.584 [0.58, 0.587]
	None	SL	0.593 [0.589, 0.596]
		SL (with screens)	0.591 [0.587, 0.594]
	SL	GLM	0.587 [0.583, 0.59]
		SL	0.586 [0.582, 0.589]
+ base set and demographic/clinical covariates	Lasso	GLM	0.589 [0.586, 0.592]
		SL	0.594 [0.591, 0.597]
	None	SL	0.608 [0.604, 0.611]
		SL (with screens)	0.604 [0.6, 0.607]
	SL	GLM	0.594 [0.591, 0.598]
		SL	0.596 [0.593, 0.6]
Lipidyzer metabolites			
+ base set of covariates	Lasso	GLM	0.523 [0.519, 0.526]
		SL	0.492 [0.488, 0.497]
	None	SL	0.528 [0.525, 0.531]
		SL (with screens)	0.518 [0.514, 0.521]
	SL	GLM	0.520 [0.517, 0.523]
		SL	0.490 [0.486, 0.495]
+ base set and demographic/clinical covariates	Lasso	GLM	0.524 [0.521, 0.528]
		SL	0.522 [0.518, 0.525]
	None	SL	0.537 [0.534, 0.541]
		SL (with screens)	0.534 [0.53, 0.537]
	SL	GLM	0.535 [0.531, 0.538]
		SL	0.528 [0.524, 0.532]
NMR metabolites			
+ base set of covariates	Lasso	GLM	0.545 [0.542,

+ base set and demographic/clinical covariates	None<br>SL<br>Lasso<br>None<br>SL	SL<br>SL<br>SL (with screens)<br>GLM<br>SL<br>GLM<br>SL<br>SL<br>SL (with screens)<br>GLM<br>SL	0.549]<br>0.547 [0.543, 0.55]<br>0.559 [0.555, 0.562]<br>0.558 [0.554, 0.561]<br>0.554 [0.551, 0.558]<br>0.554 [0.551, 0.558]<br>0.553 [0.549, 0.556]<br>0.559 [0.555, 0.562]<br>0.567 [0.563, 0.57]<br>0.567 [0.563, 0.57]<br>0.556 [0.552, 0.559]<br>0.559 [0.556, 0.563]

SUPPLEMENTAL FIGURE LEGENDS

Supplemental Figure S1.

Schematic showing the procedure taken to obtain the final sets of selected metabolites in Table 2. For each platform and set of covariates, we first perform lasso or Super Learner (SL) variable selection on each imputed dataset (for NMR, there were no missing data, so we only use the original dataset). Next, for both lasso and SL, we then determined the metabolites and covariates that were selected in at least 7 imputed datasets. Third, we combine the selected variables from the lasso and SL procedures. Our final set of variables pools across all platforms.

[1]"Covariates" denotes either base set of variables only or all covariates.

Supplemental Figure S2.

Schematic showing the procedure taken to assess prediction performance. For a given platform and set of covariates, we randomly split the data 100 times into 5-fold cross-validation (CV) allocations. Then for each of the 100 allocations separately, we performed five-fold cross-validation to assess prediction performance: for each fold k in turn, we (i) perform variable selection on the folds other than k; (ii) train a prediction model on the folds other than k; (iii) assess prediction performance on fold k; and (iv) average the prediction performance across the five folds to obtain CV prediction performance. We then averaged the CV prediction performance across the 100 random allocations to obtain the overall CV prediction performance for each platform, set of covariates, and procedure.

[1]"Covariates" denotes either base set of variables only or all covariates.

Supplemental Figure S1.

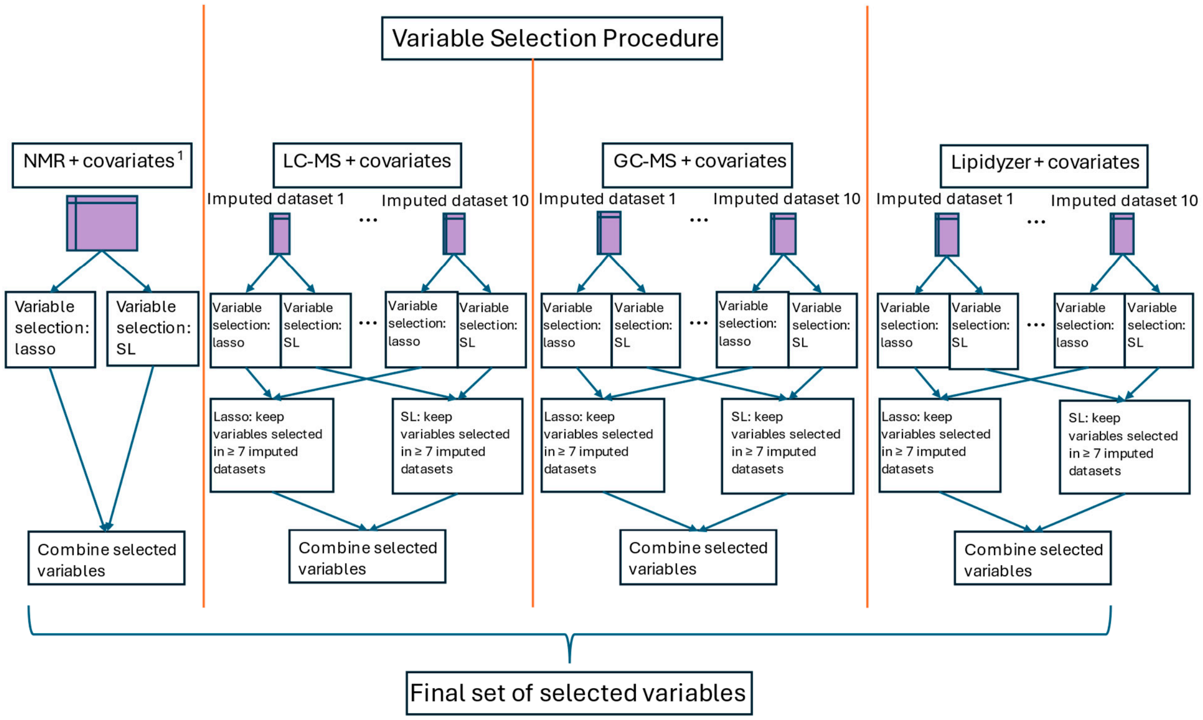

Supplemental Figure S2.

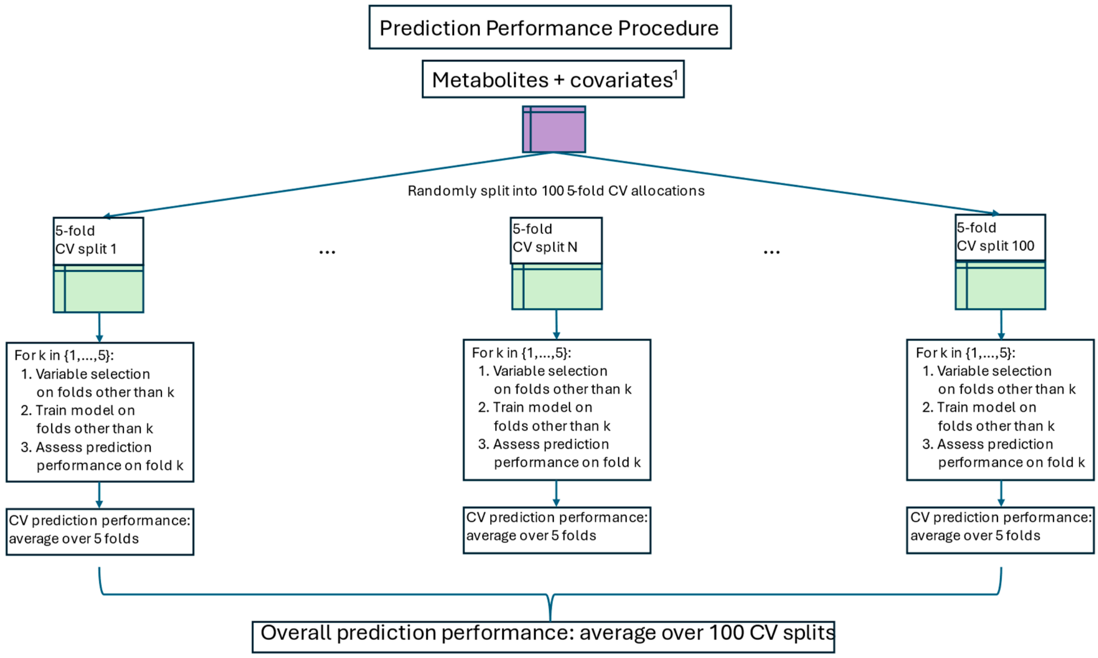

1. van Buuren, S., Groothuis-Oudshoorn, K., mice: Multivariate Imputation by Chained Equations in R. *Journal of Statistical Software* **2011,** *45* (3), 1-67.

2. van der Laan, M. J., Eric C. Polley, and Alan E. Hubbard, Super learner. *Stat. Appl. Genet. Mol. Biol.* **2007,** *6.1*.

3. Jaeger, B. C.; Cantor, R.; Sthanam, V.; Xie, R.; Kirklin, J. K.; Rudraraju, R., Improving Outcome Predictions for Patients Receiving Mechanical Circulatory Support by Optimizing Imputation of Missing Values. *Circ Cardiovasc Qual Outcomes* **2021,** *14* (9), e007071.

4. Leeb, H., Potscher, B.M., Model selection and inference: Facts and fiction. *Econometric Theory* **2005,** *21*.

5. Polley, E., LeDell, E, Kennedy, C, and van der Laan, MJ. SuperLearner: Super Learner Prediction. R package version 2.0-26. 2023. https://github.com/ecpolley/SuperLearner.
